# Obesity and total joint arthroplasty: Does weight loss in the preoperative period improve perioperative outcomes?

**DOI:** 10.1186/s42836-022-00149-0

**Published:** 2022-11-04

**Authors:** Jacob Laperche, Richard Feinn, Karen Myrick, Mohamad J. Halawi

**Affiliations:** 1 Frank H. Netter School of Medicine, Quinnipiac University, North Haven, CT 06473 USA; 2 Department of Nursing, School of Interdisciplinary Health and Science, University of Saint Joseph, West Hartford, CT 06117 USA; 3 Department of Orthopedic Surgery, Baylor College of Medicine, 7200 Cambridge Street, Suite 10A, Houston, TX 77030 USA

**Keywords:** Obesity, Weight loss, Total hip arthroplasty, Total knee arthroplasty, Outcomes

## Abstract

**Background:**

The obese population is more likely to develop degenerative joint disease requiring total joint arthroplasty (TJA) and also experience increased rates of adverse post-surgical outcomes. This study assessed whether a quantifiable weight loss prior to TJA had any impact on perioperative and 30-day outcomes in obese patients.

**Method:**

Using the American College of Surgeons-National Surgical Quality Improvement Program database, obese patients who underwent total hip or total knee arthroplasty and lost at least 10% of their total body weight prior to surgery were identified and matched to other obese individuals undergoing the same procedures without weight loss. Perioperative outcomes, including operative time, length of stay, discharge destination, or 30-day adverse events, including complications, re-admissions, re-operations, and mortality, were then compared using conditional Logistic regression analysis.

**Results:**

Analysis showed no difference between the two groups in terms of operative time, length of stay, discharge destination, or 30-day adverse events, including complications, re-admissions, re-operations, and mortality.

**Conclusion:**

The results of this study suggest that weight loss alone in the preoperative period may not be sufficient to mitigate the effects of obesity on immediate post-TJA outcomes.

## Introduction

Obesity has been well established to be a significant risk factor for lower post-surgical outcomes. Within total joint arthroplasty (TJA), obesity has been associated with higher rates of impaired wound healing, infection, deep venous thrombosis, prolonged hospitalization, and revision surgery [1–6]. Consequently, strategies to improve TJA outcomes in obese patients are vital, especially with an estimated 12.5% of the US population considered obese [7].

Among the most common strategies to mitigate the effects of obesity are preoperative weight loss and the implementation of body mass index cutoffs [6, 8–10]. The benefits of these recommendations continue to be debated as the ability of weight loss to improve postoperative outcomes remains unclear. Since weight loss can be a challenging task for many patients, it is important for providers to demonstrate tangible improvements in order to justify such weight loss and effectively motivate patients to lose weight.

The objective of this preliminary study was to assess the extent to which weight loss has on a number of perioperative outcomes in obese patients undergoing primary, unilateral TJA. These include operative time, hospital length of stay, discharge destination, and 30-day complications, re-operations, re-admissions, and mortality. We hypothesized that weight loss in obese patients would improve those outcomes. A better understanding of the effect of weight loss in TJA patients is critical as obesity rates and the demand for TJA continue to increase. The information is needed in order to treat these patients more effectively and optimize their outcomes.

## Methods

### Patients

A retrospective review of the American College of Surgeons-National Surgical Quality Improvement Program (ACS-NSQIP) database was performed. All data were non-identifiable, making the review IRB exempt. Obese patients, defined as having a body mass index (BMI) ≥ 30, who underwent primary, unilateral total hip or knee arthroplasty (THA, TKA) from the years 2010–2018 were identified. Two groups were compared: a weight loss group and a non-weight loss group. The weight loss group was defined as individuals with a BMI ≥ 30 who had a minimum of 10% reduction in their total body weight in the 6 months prior to surgery. Weight loss status was indicated as a variable in the ACS-NSQIP database. The non-weight loss group had a BMI ≥ 30 but without significant weight loss.

### Measures and outcomes

Preoperative characteristics of the patients included surgery type (THA or TKA), age, sex, race, BMI, functional status (dependent on another individual for care or not), point of origin other than home, smoking or tobacco use within one year from surgery, chronic steroid use, diabetes, hypertension, chronic obstructive pulmonary disease (COPD), congestive heart failure (CHF), bleeding disorders, serum albumin levels, dyspnea, and American Society of Anesthesiologists (ASA) Physical Status Classification. Patients were evaluated and compared in terms of the following outcomes: operative time (minutes), length of hospital stay (LOS, days), discharge to rehabilitation or skilled nursing facilities, and 30-day complications (including superficial surgical site infection [SSI], deep incision SSI, organ space SSI, wound disruption, pneumonia, unplanned intubation, pulmonary embolism, ventilator for >48 hours, progressive renal insufficiency, acute renal failure, urinary tract infection, cerebrovascular accident or stroke with neurologic deficit, coma >24 hours, peripheral nerve injury, cardiac arrest requiring CPR, myocardial infarction, bleeding requiring transfusion, prosthesis loosening, deep vein thrombosis, thrombophlebitis, sepsis, or septic shock), re-admissions, re-operations, and mortality.

### Statistical analysis

Descriptive statistics included means with standard deviations for continuous variables, and frequencies with percentages for categorical variables. To test if weight loss affected surgical outcomes, a conditional Logistic regression model was made using the stratified Cox regression procedure. Patients who lost 10% or more of their body weight were matched with patients who did not lose 10% or more of their body weight (1:2 when possible). The criteria for matching were the type of procedure (THA or TKA), year of surgery, age, sex, functional status, and American Society of Anesthesiologists Physical Status Classification. Patients were matched exactly on all variables except age, where controls had to be within one year of the cases. Furthermore, a propensity score was created using the remaining baseline variables and entered into the Cox regression model as a covariate. Models were run separately for THA and TKA patients. Analyses were conducted by using SAS v9.4 and statistical significance was defined as *P* < 0.05.

## Results

### Patient characteristics

A total of 315,000 obese patients had received either TKA or THA. Of them, 302 obese patients with ≥ 10% weight loss were matched with 567 obese patients who did not lose weight (265 cases matched with two controls and 37 matched with one control) (Table 1). Among all study patients, 21% had a BMI over 40 and were considered morbidly obese. While the variables used for matching did not differ between the two groups, there were differences in other variables. In particular, when compared to patients who did not lose weight, patients who lost weight had higher rates of chronic steroid use (7% *vs*. 4%, *P* = 0.029), diabetes (26% *vs*. 18%, *P* = 0.004), COPD (8% *vs*. 4%, *P* = 0.008), CHF (3% *vs*. 1%, *P* = 0.009), and lower serum albumin (mean of 3.90 *vs*. 4.08, *P* < 0.001).

**Table 1:** Patient characteristics of the study groups

	**BMI 30** ≥ **and No Weight Loss**	**BMI** ≥ **30 and Weight Loss**	***P* Value**
*n*	567	302	
Surgery			
Total Hip Arthroplasty	218 (38%)	115 (38%)	
Total Knee Arthroplasty	349 (62%)	187 (62%)	
Age (years)	63.6 ± 9.4	63.6 ± 9.4	0.932
Sex (female)	348 (61%)	182 (60%)	0.749
Race (Caucasian)	462 (82%)	248 (82%)	0.974
Body mass index	36.7 ± 5.6	36.5 ± 5.4	0.712
Functional status (Dependent)	23 (4%)	14 (4%)	0.861
Point of origin other than home	2 (<1%)	4 (1%)	0.099
Smoker within 1 year	53 (9%)	32 (11%)	0.555
Chronic steroid use	22 (4%)	22 (7%)	0.029
Diabetes	101 (18%)	79 (26%)	0.004
Hypertension	389 (69%)	221 (73%)	0.161
Chronic obstructive pulmonary disease	20 (4%)	23 (8%)	0.008
Congestive heart failure	4 (1%)	9 (3%)	0.009
Bleeding disorders	18 (3%)	14 (5%)	0.276
Serum albumin level (g/dL)	4.08 ± 0.37	3.90 ± 0.52	<0.001
Dyspnea	45 (8%)	35 (12%)	0.076
American Society of Anesthesiologists Physical Status Classification	359 (63%)	193 (64%)	0.998

### Regression analysis

Table 2 shows the results for the conditional Logistic regression with the propensity score adjustment separately for THA and TKA. After controlling for all baseline differences, the two groups were statistically similar in all the study outcomes (*P* > 0.05): operative time, LOS, discharge destination, 30-day complications, re-admissions, re-operations, and mortality.

**Table 2:** Multivariate regression analysis for total hip and knee arthroplasty

	**Hip (*n*=333)**			**Knee (*n*=536)**		
	Odds Ratio	95% CI	*P*-Value	Odds Ratio	95% CI	*P*-Value
Operation Time >100 Minutes	1.03	0.70–1.52	0.890	0.97	0.70–1.33	0.826
Operation Time	1.04	0.96–1.16	0.414	1.00	0.93–1.08	0.958
Any 30-day Complications	1.08	0.83–1.40	0.557	0.97	0.79–1.20	0.772
Length of Stay (Days)	1.40	0.85–2.29	0.184	0.93	0.83–1.05	0.224
Discharge to Rehab/Skilled care	1.50	0.62–3.64	0.370	0.83	0.42–1.66	0.604
Re-operation within 30 days	1.54	0.40–6.03	0.532	0.33	0.04–3.01	0.325
Re-admission within 30 days	0.70	0.22–2.19	0.535	0.70	0.18–2.77	0.613
Any Surgical Complications	1.42	0.80–2.51	0.234	0.88	0.47–1.64	0.686
Any Medical Complications	1.00	0.76–1.32	1.00	1.01	0.81–1.25	0.962

## Discussion

The fact that obese patients often encounter more frequent post-surgical complications have led many to recommend weight loss prior to TJA but the results are mixed [9, 11–14]. In this study, we sought to investigate the effect of weight loss on immediate post-TJA outcomes. Contrary to our hypothesis, we found that reduction of at least 10% of total body weight in obese patients prior to surgery had no significant impact on operative time, LOS, discharge destination as well as 30-day complications, re-admissions, re-operations, and mortality when compared to their counterparts who did not lose weight. These findings were consistent for both THA and TKA.

Our findings appear to be consistent with studies showing that preoperative weight did not reduce complications. Inacio *et al*. [15] retrospectively reviewed 1332 TKAs and 732 THAs in obese patients from a single health care system who had a 5% decrease in their weight prior to surgery. At one year follow-up, no difference was found in the rates of surgical site infections or re-admissions compared to patients who had no weight loss prior to surgery. In a meta-analysis by Smith *et al*. [16], THA and TKA complication rates in 657 obese patients who underwent bariatric surgery were compared to 22,691 obese patients who did not. There was no difference in the study outcomes including infections, revisions, deep vein thrombosis, pulmonary embolism, or mortality. The authors concluded that bariatric surgery before THA or TKA did not reduce complications or improve outcomes. The lack of evidence for weight loss to improve complication rates and outcomes in obese patients perhaps warrants exploration for alternative measures in addition to weight loss alone.

The lack of improvements in outcomes may be two-fold. The weight reduction may not have been enough to impact outcomes or not enough time following weight reduction was permitted. In this study, weight loss was defined as at least a 10% reduction in total body weight. It is possible that a more significant weight reduction may be needed to effect benefits. Weight loss prior to surgery may also require some time for the body to recover from the induced catabolic state before the benefits of weight loss are seen. As weight loss captured in our study occurred in the 6 months prior to surgery, it is possible that such weight loss was too quick and too close to surgery.

Operative time can be increased with obese patients due to the need for large exposure and tissue dissection, and thus it has been implicated as a contributing factor to increased complications in this patient population [17]. Our study however did not identify any differences in operative time. While a reduction in weight of 10% of total body weight is a significant amount, it may not provide a significant reduction in total tissue that needs to be dissected compared to non-weight loss obese patients. Additionally, if weight loss occurred primarily in the abdomen or viscera, the reduction would likely not impact knee or hip procedures significantly. A 2017 study found that among 180 overweight and obese patients who underwent a 22-week weight loss program through diet and exercise, men lost the highest percentage of weight from the trunk while women lost the highest percentage of weight from the hips and legs [18]. This finding indicates a difference in sex may also play a role in weight loss reducing operative time and it may depend on the operation planned. The similar operative time recorded between our study groups, and the lack of stratification by sex, may explain their similar complication rates.

Obesity has been shown to increase hospital LOS and the likelihood of discharge to a rehabilitation facility following TJA [19, 20]. Length of stay and discharge destination have become important quality metrics in our evolving value-based healthcare system as shortened LOS and direct discharge to home have been shown to have significant cost-saving effects [21, 22]. Cytokines such as IL-1β, IL-6 and TNF-α have been implicated in inflammation and pain. One study looking at these levels following weight loss surgery found a postoperative decrease in IL-6 and TNF-α [23]. Weight loss prior to surgery could also show a similar decrease in proinflammatory cytokines and a possible decrease in pain postoperatively, which could have an added benefit of decreased LOS. The results of this study did not show weight loss alone to be sufficient to significantly reduce LOS and non-home discharge rates.

Limitations of this study include the possibility of low power to adequately identify true differences between these groups. Due to the variable outcomes and a limited number of patients with at least 10% weight loss, we included all those patients and compared them with the non-weight loss cohort stringently matched by a number of baseline factors. Second, it was not reported in the database if weight loss was intentional, the result of diet and exercise or surgery, or unintentional, potentially due to the disease process. This distinction would be important in making a recommendation to patients. Third, the absolute amount of weight loss was not recorded either in the database. Instead, we relied on the reported binary variable indicating at least 10% weight loss or not. It’s possible the actual weight loss amount, rather than the percentage, could be a better indicator. Fourth, weight loss is only one strategy to optimize outcomes and perhaps should not be looked at *in silo*. For example, a myriad of other factors known to impact perioperative outcomes should also be taken into consideration as part of performing a successful total joint replacement, such as nasal staphylococcal decolonization, smoking cessation, ensuring adequate nutritional status, and optimization of medical comorbidities.

## Conclusion

In conclusion, this retrospective review of a national quality database showed that a 10% reduction of the total body weight in the 6 months prior to surgery did not improve the perioperative outcomes in obese patients undergoing primary, unilateral TJA. Specifically, there were no reduction in operative time, LOS, non-home discharge, and 30-day adverse events.

## Data Availability

The data analyzed for this study can be found at the American College of Surgeons-National Surgical Quality Improvement Program.

## Abbreviations

TJA: Total Joint Arthroplasty
ACS-NSQIP: American College of Surgeons-National Surgical Quality Improvement Program
THA: Total Hip Arthroplasty
TKA: Total Knee Arthroplasty
BMI: Body Mass Index
ASA: American Society of Anesthesiologists
SSI: Superficial Surgical Site Infection
LOS: Length of Stay

## References

[1] Haynes J, Nam D, Barrack RL (2017). Obesity in total hip arthroplasty: does it make a difference?. Bone Joint J.

[2] Jeschke E, Citak M, Günster C, Halder AM, Heller KD, Malzahn J (2018). Obesity increases the risk of postoperative complications and revision rates following primary total hip arthroplasty: an analysis of 131,576 total hip arthroplasty cases. J Arthroplasty.

[3] Martin JR, Jennings JM, Dennis DA (2017). Morbid obesity and total knee arthroplasty: a growing problem. J Am Acad Orthop Surg.

[4] Roth A, Khlopas A, George J, Churchill JL, Molloy R, Mont MA (2019). The effect of body mass index on 30-day complications after revision total hip and knee arthroplasty. J Arthroplasty.

[5] Sloan M, Sheth N, Lee GC (2019). Is obesity associated with increased risk of deep vein thrombosis or pulmonary embolism after hip and knee arthroplasty? A large database study. Clin Orthop Relat Res.

[6] Springer BD, Carter JT, McLawhorn AS, Scharf K, Roslin M, Kallies KJ (2017). Obesity and the role of bariatric surgery in the surgical management of osteoarthritis of the hip and knee: a review of the literature. Surgery for Obesity and Related Diseases.

[7] Chooi YC, Ding C, Magkos F (2019). The epidemiology of obesity. Metabolism.

[8] Gandler N, Simmance N, Keenan J, Choong PF, Dowsey MM (2016). A pilot study investigating dietetic weight loss interventions and 12 month functional outcomes of patients undergoing total joint replacement. Obes Res Clin Pract.

[9] Godziuk K, Prado CM, Beaupre L, Jones CA, Werle JR, Forhan M (2021). A critical review of weight loss recommendations before total knee arthroplasty. Joint Bone Spine.

[10] Milla C, Lo Menzo E, Morton J, Rosenthal RJ (2019). Obesity disease pandemic on joint disease and longevity. J Arthroplasty.

[11] Ast MP, Abdel MP, Lee YY, Lyman S, Ruel AV, Westrich GH (2015). Weight changes after total hip or knee arthroplasty: prevalence, predictors, and effects on outcomes. J Bone Joint Surg Am.

[12] Murr MM, Streiff WJ, Ndindjock R (2021). A literature review and summary recommendations of the impact of bariatric surgery on orthopedic outcomes. Obes Surg.

[13] Nearing EE, Santos TM, Topolski MS, Borgert AJ, Kallies KJ, Kothari SN (2017). Benefits of bariatric surgery before elective total joint arthroplasty: is there a role for weight loss optimization?. Surg Obes Relat Dis.

[14] Seward MW, Antonelli BJ, Giunta N, Iorio R, Fitz W, Lange JK (2020). Weight loss before total joint arthroplasty using a remote dietitian and mobile app: study protocol for a multicenter randomized, controlled trial. J Orthop Surg Res.

[15] Inacio MC, Kritz-Silverstein D, Raman R, Macera CA, Nichols JF, Shaffer RA (2014). The impact of pre-operative weight loss on incidence of surgical site infection and readmission rates after total joint arthroplasty. J Arthroplasty.

[16] Smith TO, Aboelmagd T, Hing CB, MacGregor A (2016). Does bariatric surgery prior to total hip or knee arthroplasty reduce post-operative complications and improve clinical outcomes for obese patients? Systematic review and meta-analysis. Bone Joint J.

[17] Chen K, Pan Y, Zhai ST, Cai JQ, Chen QL, Chen DW (2017). Laparoscopic gastrectomy in obese gastric cancer patients: a comparative study with non-obese patients and evaluation of difference in laparoscopic methods. BMC Gastroenterol.

[18] Benito PJ, Cupeiro R, Peinado AB, Rojo MA, Maffulli N, PRONAF Study Group (2017). Influence of previous body mass index and sex on regional fat changes in a weight loss intervention. Phys Sportsmed.

[19] Childs BR, Nahm NJ, Dolenc AJ, Vallier HA (2015). Obesity is associated with more complications and longer hospital stays after orthopaedic trauma. J Orthop Trauma.

[20] O'Connell BP, Rizk HG, Stevens SM, Nguyen SA, Meyer TA (2015). The relation between obesity and hospital length of stay after elective lateral skull base surgery: an analysis of the American college of surgeons national surgical quality improvement program. ORL J Otorhinolaryngol Relat Spec.

[21] Werner RM, Coe NB, Qi M, Konetzka RT (2019). Patient outcomes after hospital discharge to home with home health care vs to a skilled nursing facility. JAMA Intern Med.

[22] Gwam CU, Mohamed NS, Dávila Castrodad IM, George NE, Remily EA, Wilkie WA (2020). Factors associated with non-home discharge after total knee arthroplasty: potential for cost savings?. Knee.

[23] Cottam D, Fisher B, Ziemba A, Atkinson J, Grace B, Ward DC (2010). Tumor growth factor expression in obesity and changes in expression with weight loss: another cause of increased virulence and incidence of cancer in obesity. Surg Obes Relat Dis.

